# Supplementary material for: Structures of LIG1 that engage with mutagenic mismatches inserted by polβ in base excision repair
Source: Nat Commun. 2022 Jul 5;13:3860. doi: 10.1038/s41467-022-31585-w (PMC9256674; doi:10.1038/s41467-022-31585-w)
Supplement: Supplementary file 1 — Supplementary Information [file 41467_2022_31585_MOESM1_ESM.pdf]

SUPPLEMENTARY INFORMATION

Structures of LIG1 that engage with mutagenic mismatches inserted by polβ in base excision repair

Qun Tang[1], Mitchell Gulkis[1], Robert McKenna[1] and Melike Çağlayan[1,*]

[1]Department of Biochemistry and Molecular Biology, University of Florida, Gainesville, FL 32610, USA

*To whom correspondence should be addressed. Tel.: +1 352-294-8383; Email: caglayanm@ufl.edu

Supplementary Figures 1-15

Supplementary Tables 1-7

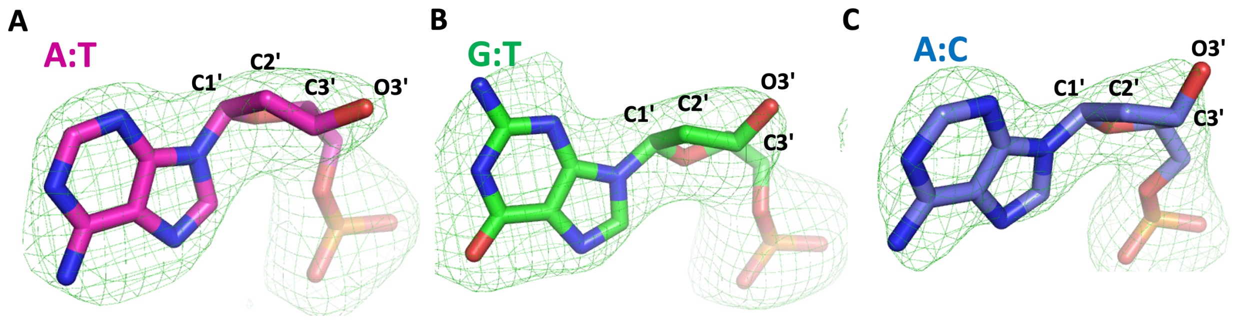

Supplementary Fig. 1: LIG1/mismatch structures and the sugar conformation. Fo - Fc omit electron density maps (3σ) of LIG1 A:T (**A**), G:T (**B**) and A:C (**C**) structures show the differences in the angle between C1, C2, C3, and O3.

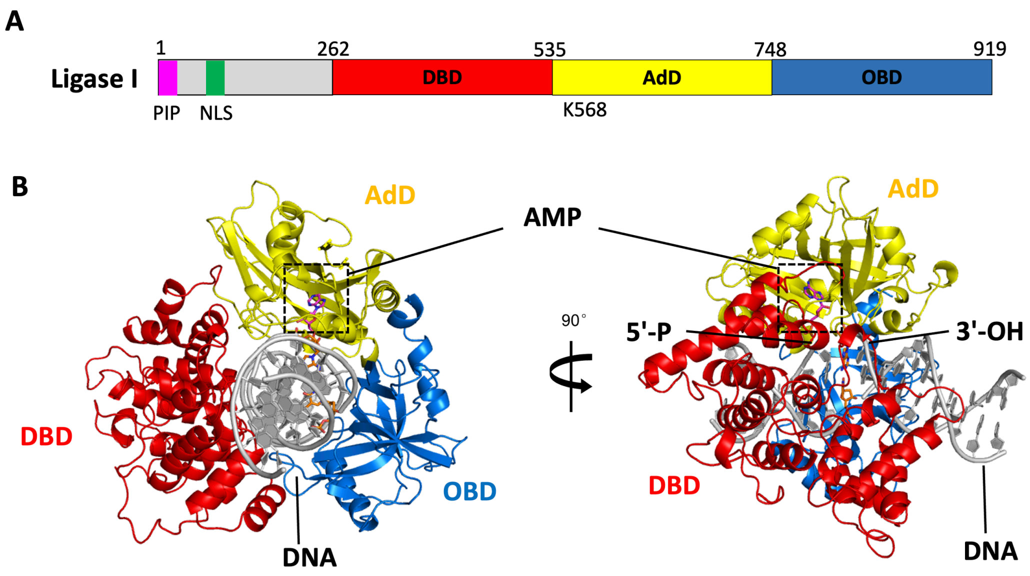

Supplementary Fig. 2: Schematic and structural views of LIG1. A Human DNA ligase I protein contains the N-terminal region (amino acids 1-262), which is missing from the crystal structure, and includes the PCNA interacting peptide (PIP) and a nuclear localization signal (NLS). This noncatalytic part of LIG1 mediates its interaction with DNA-sliding clamps and polβ. The catalytic core of LIG1 consists of the Adenylation (AdD, yellow) and OB-fold (OBD, blue) domains. K568 refers to an active site lysine that is adenylated by ATP during the first step of ligation reaction. Human LIG1 harbors the DNA binding domain (DBD, red) that stabilizes the DNA and interacts with the minor groove of the nick DNA. OBD makes a ring-shaped protein structure that encircles the DNA during nick sealing. **B** LIG1 (cartoon) encircling nick DNA is depicted in complex with AMP-DNA for cognate A:T base pair and AMP-LIG1 for A:C mismatch. AMP (orange) and DNA (grey) are shown as sticks at 3'-OH of nick (magenta).

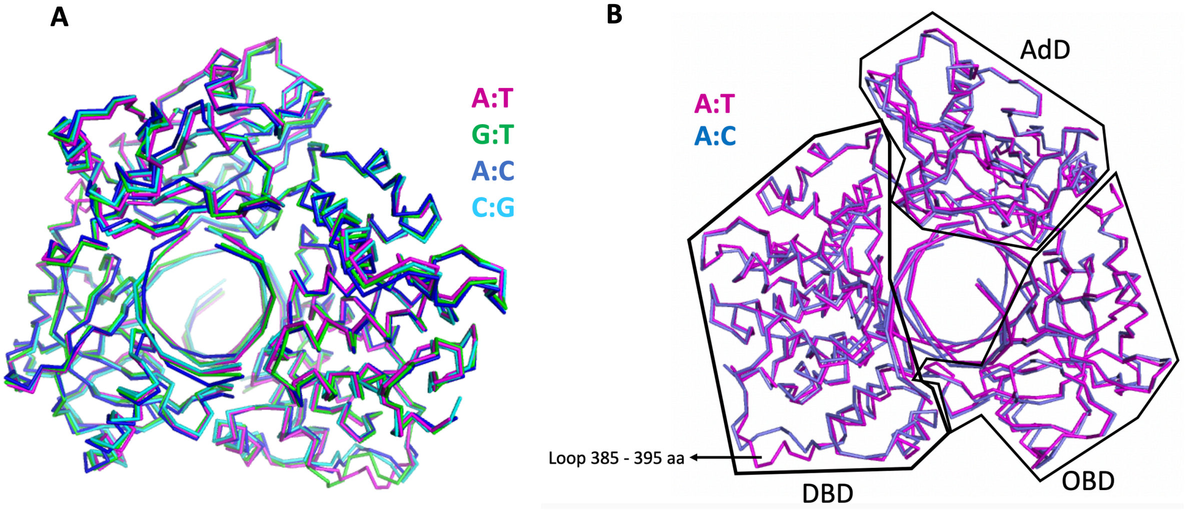

Supplementary Fig. 3: Structural domains of LIG1 encircling nick DNA complexes with mismatched or cognate base-paired ends. A Overlay of LIG1 structures for wild-type LIG1/nick DNA duplex containing C:G (PDB: 6P09, cyan) and LIG1 EE/AA/nick DNA duplexes containing A:T (magenta) or mismatches G:T (green) and A:C (blue). **B** Overlay of LIG1/A:T (magenta) and LIG1/A:C (blue) shows the structural composition of all three domains of the protein with a difference in the loop region consisting of amino acids 385-395. All structures are shown as ribbons.

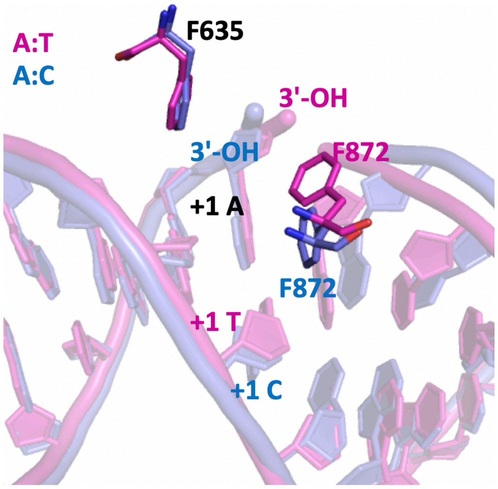

Supplementary Fig. 4: Positions of F635 and F872 residues in the LIG1/A:T and A:C structures. Overlay of the LIG1 structures bound to the nick DNA duplexes containing cognate A:T (magenta) and mismatch A:C (blue) shows the shift and no conformational change in the positions of F872 and F635 residues (stick), respectively.

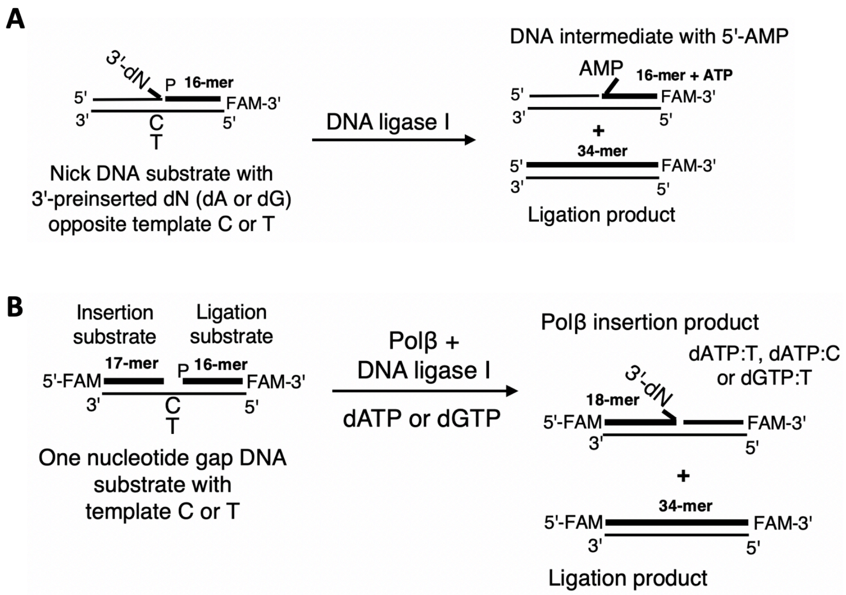

Supplementary Fig. 5: Illustrations of DNA repair assays performed in this study. **A** Ligation assays were used to evaluate the substrate specificity of LIG1 for the nick DNA substrates including 3'-preinserted mismatches dG:T or dA:C and cognate dA:T base pair. **B** Coupled assays were used to evaluate the ligation of polβ correct (dGTP:C) or mismatch (dGTP:T or dATP:C) insertions in the reaction mixture including both polβ and LIG1 using one nucleotide gap DNA substrates with template base C or T.

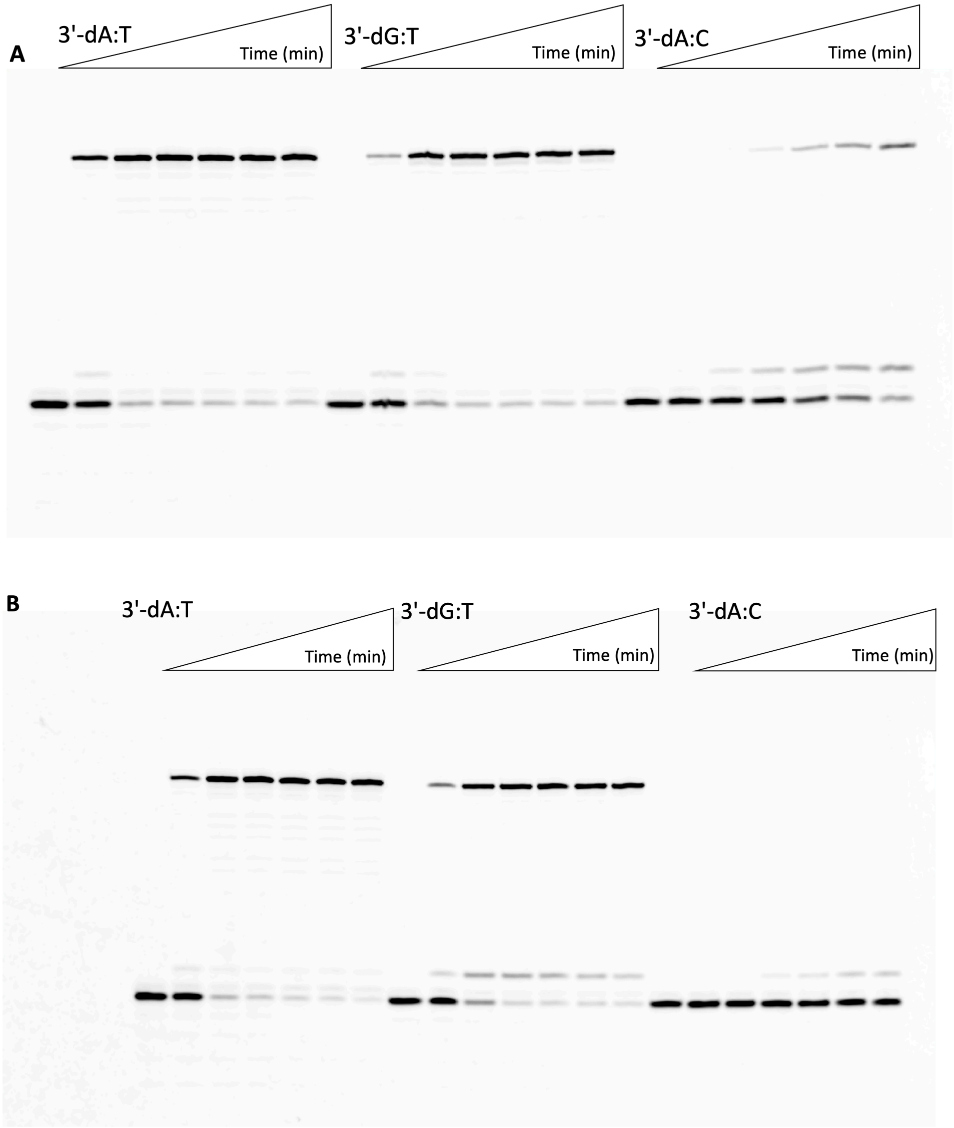

Supplementary Fig. 6: Uncropped gel images showing the ligation of nick DNA substrates with 3'-preinserted dA:T, dG:T, and dA:C by EE/AA (**A**) and wild-type (**B**) LIG1. The results are presented in Fig. 6.

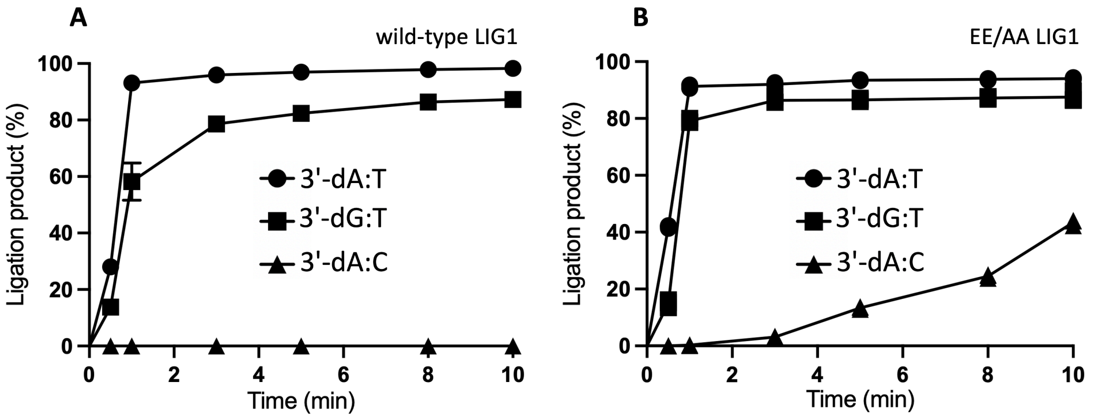

Supplementary Fig. 7: Ligation efficiencies of mismatch-containing nick DNA substrates by LIG1. Plots show time-dependent changes in the amount of ligation products by wild-type (**A**) and EE/AA (**B**) of LIG1. The data represent the average of three independent experiments ± SD.

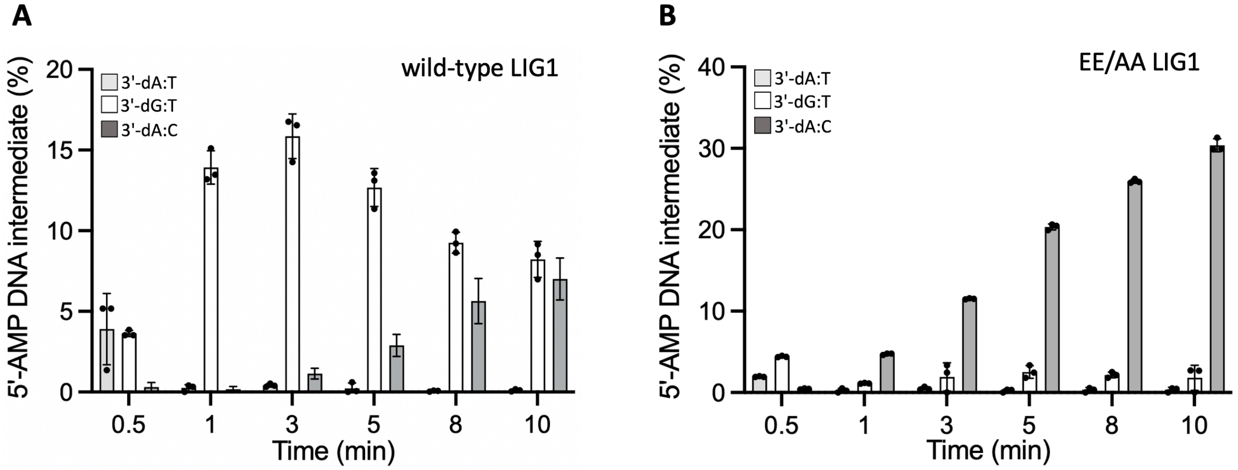

Supplementary Fig. 8. Formation of DNA intermediates with 5'-AMP during the nick sealing of mismatches by LIG1. Graphs show time-dependent changes in the amount of DNA intermediates by wild-type (**A**) and EE/AA (**B**) of LIG1 for the nick DNA substrates with 3'-preinserted dA:T, dG:T, and dA:C. The data represent the average of three independent experiments ± SD.

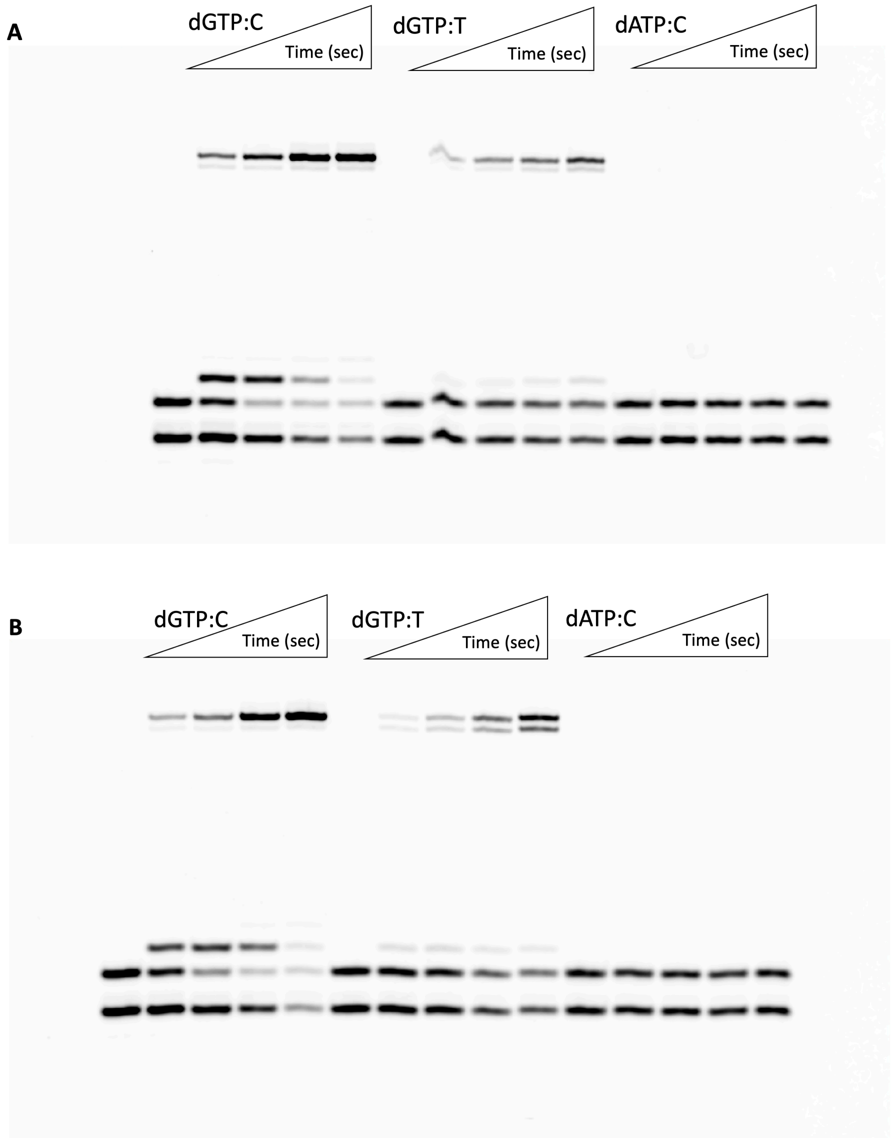

Supplementary Fig. 9: Uncropped gel images showing the ligation of polβ mismatch nucleotide insertion products by wild-type (**A**) and EE/AA (**B**) of LIG1. The results are presented in Fig. 7.

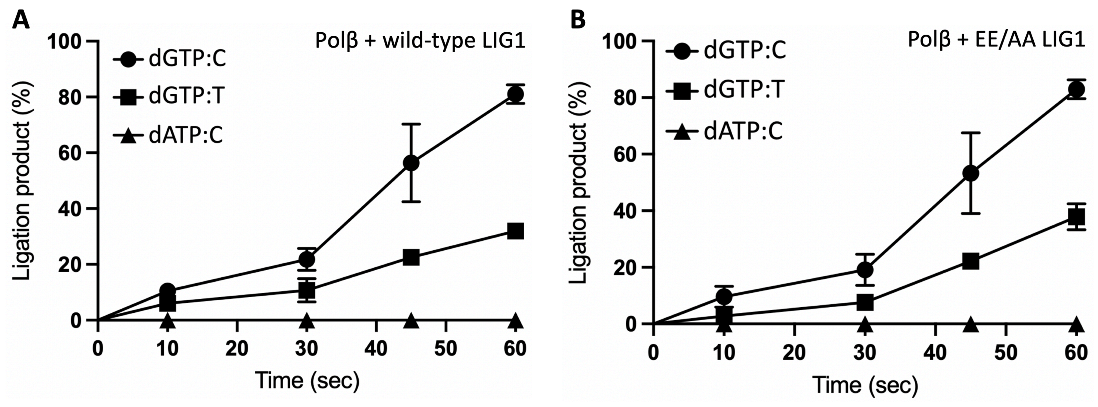

Supplementary Fig. 10: Ligation of polβ mismatch nucleotide insertion products by LIG1. Plots show time-dependent changes in the amount of ligation products for polβ correct dGTP:C and mismatched dGTP:T and dATP:C insertions coupled to DNA ligation by wild-type (**A**) and EE/AA (**B**) of LIG1. The data represent the average of three independent experiments ± SD. The results are presented in Fig. 7.

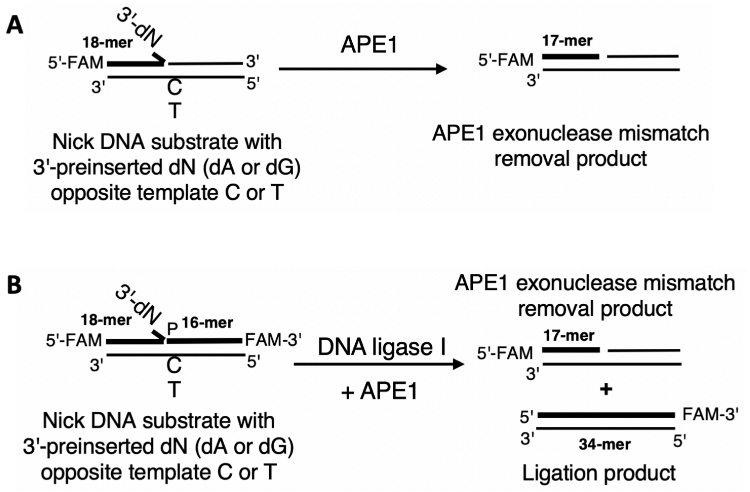

Supplementary Fig. 11: Illustrations of APE1 experiments performed in this study. **A** APE1 exonuclease activity assays were used to evaluate the removal of a mismatched base from the nick DNA substrates including 3'-preinserted mismatches G:T or A:C. **B** Repair assays were used to evaluate the ligation coupled to removal of 3'-mismatches in the reaction mixture including both APE1 and LIG1 using the nick DNA substrates including 3'-preinserted mismatches G:T or A:C.

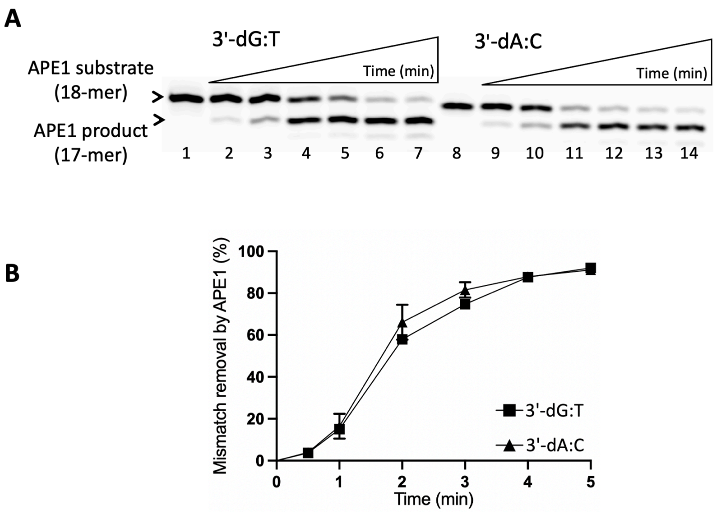

Supplementary Fig. 12: APE1 mismatch removal from nick repair intermediates with mismatches. **A** Lanes 1 and 8 are the negative enzyme controls of the nick DNA substrates with 3'-preinserted dG:T and dA:C, respectively. Lanes 2-7 and 9-14 are the reaction products of mismatch removal by APE1 from the nick DNA substrates containing 3'-preinserted dG:T and dA:C mismatches, respectively, and correspond to time points of 0.5, 1, 2, 3, 4, and 5 min. **B** Plot shows time-dependent changes in the amount of APE1 mismatch removal products. The data represent the average of three independent experiments ± SD.

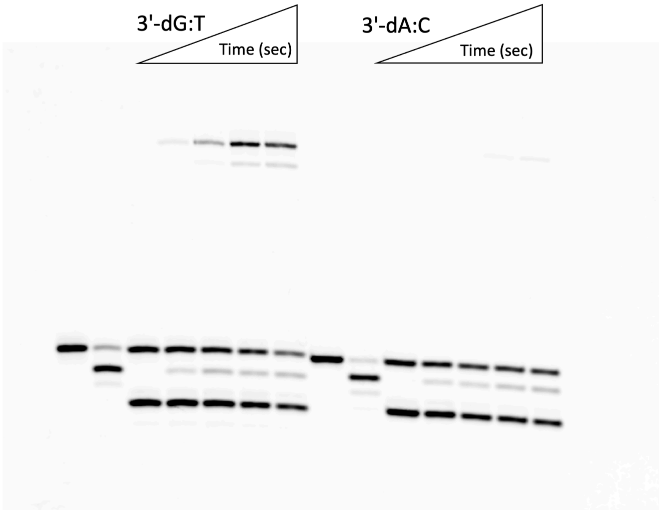

Supplementary Fig. 13: Uncropped gel image showing the ligation of mismatch-containing nick DNA substrates by LIG1 and the removal of 3'-mismatched bases by APE1. The results are presented in Fig. 8.

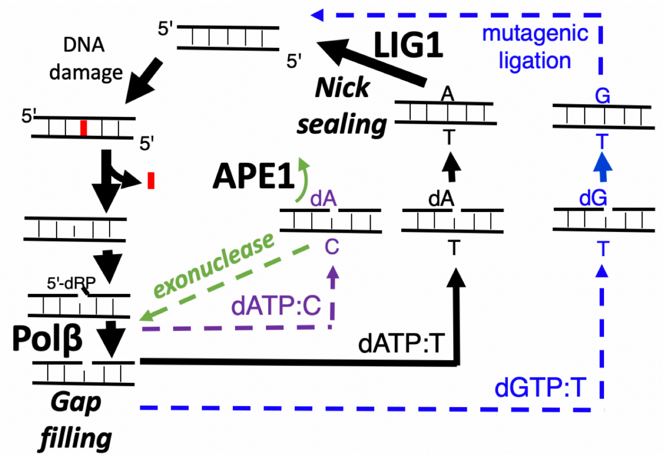

Supplementary Fig. 14: Illustration of the downstream steps involving polβ gap filling and subsequent nick sealing in the BER pathway. The model shows the efficient nick sealing of the repair intermediate after polβ dATP:T insertion. Polβ dGTP insertion opposite T results in the mutagenic ligation of nick repair intermediate with G:T mismatch by LIG1. In contrast, the nick repair intermediate with a mismatched A:C ends can serve as a structural fidelity checkpoint and a signal for proofreading enzyme such as APE1 for a mismatched base removal.

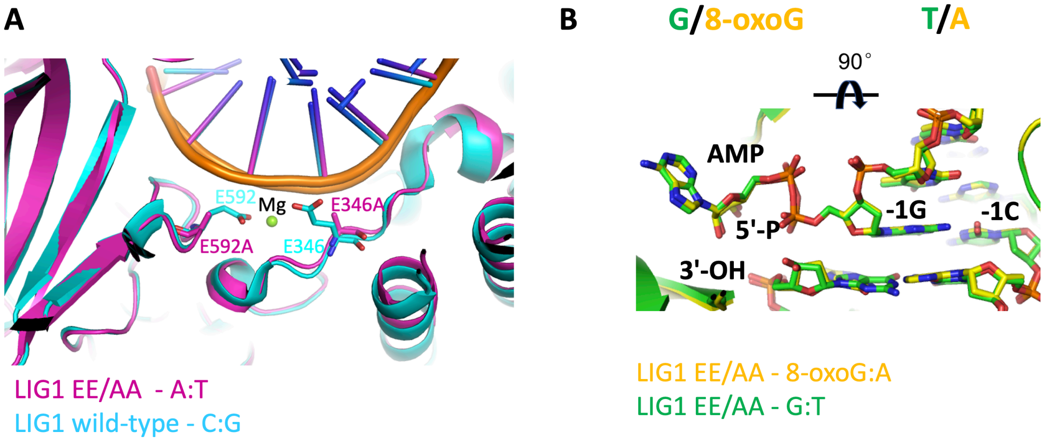

Supplementary Fig. 15: The comparison of LIG1 wild-type and EE/AA mutant structures in complex with the nick DNA containing mismatched (G:T), damaged (8-oxoG:A), or matched (A:T or C:G) ends. **A** Superimposition of previously solved wild-type LIG1/C:G (PDB: 6P09, cyan) with our EE/AA LIG1/A:T (magenta) structure is shown as cartoon and the amino acid residues E346 and E592 (Mg^{HiFi} site) that are mutated in LIG1 EE/AA mutant are shown as sticks. Mg^{2+} (green) is shown as sphere. **B** Superimposition of our EE/AA LIG1/G:T (green) with previously solved EE/AA LIG1/8-oxoG:A (PDB: 6P0E, yellow) shows differences in the positions of 5'-P with AMP and -1G and -1C nucleotides relative of the upstream DNA.

DNA Polymerase	Family	Mismatch	Location	Conformation	Reference
BF	A	dG:T	n-1 and n-2	Wobble	Johnson and Beese (2004)
BF	A	dG:T	n-3 and n-4	Ionized or tautomeric	Johnson and Beese (2004)
BF	A	dG:T	n-6	Wobble	Johnson and Beese (2004)
BF	A	dT:G	n-1	Wobble	Johnson and Beese (2004)
BF	A	dC:A	Insertion site (Mg2+)	Wobble	Wang et al (2011)
BF	A	dC:A	Insertion site (Mn2+)	WC-like	Wang et al (2011)
BF	A	dC:A	Post-insertion site, n-4, n-6	Wobble	Wang et al (2011)
BF	A	dC:A	n-3	WC-like	Wang et al (2011)
RB69	B	dG:T	n-1 and n-4	Wobble	Xie and Konigsberg (2014)
RB69	B	dG:T	n-2 and n-3	WC-like	Xie and Konigsberg (2014)
RB69	B	dG:T	n-5	Reverse Wobble	Xie and Konigsberg (2014)
RB69	B	dT:G	n-1, n-3, n-4, and n-5	Wobble	Xie and Konigsberg (2014)
RB69	B	dT:G	n-2	WC-like	Xie and Konigsberg (2014)
Pol β	X	dG:T	Primer Terminus (Binary Complex)	Wobble	Batra et al (2016)
Pol β	X	dG:T	Primer Terminus (Ternary Complex)	Weak Hoogsteen	Batra et al (2016)
Pol β	X	dA:C	Insertion site (Mn2+)	Staggered	Batra et al (2008)
Pol β	X	dT:G	Insertion site (Mg2+)	Pseudo-propeller twist	Koag et al (2014)
Pol β	X	dT:G	Insertion site (Mn2+)	WC-like	Koag et al (2014)
Pol β	X	dC:A	Insertion site (Mg2+)	Staggered	Koag et al (2014)
Pol β	X	dC:A	Insertion site (Mn2+)	Staggered	Koag et al (2014)
Pol λ	X	dG:T	Insertion site	WC-like	Bebenek et al (2011)
Pol λ	X	dG:T	Primer Terminus	Wobble	Bebenek et al (2011)
Pol μ	X	dG:T	Insertion site (Reaction ready)	WC-like	Guo et al (2021)
Pol μ	X	dG:T	Insertion site (Flipped conformation)	WC-like	Guo et al (2021)
Pol μ	X	dG:T	Insertion site (Active site double mutant)	Wobble	Guo et al (2021)
Dpo4	Y	dG:T	Primer Terminus	Reverse Wobble (but authors hypothesize that it alternates between reverse and normal wobble)	Trincao et al (2004)
Dpo4	Y	dG:T	Insertion site	Wobble/Non-coplanar	Vaisman et al (2005)
Dpo4	Y	dG:T	Insertion site (after nucleotide addition)	Non-coplanar	Vaisman et al (2005)
Pol η	Y	dG:T	Insertion site (with several different primer terminus bps)	Wobble	Zhao et al (2013)

Supplementary Table 1: Comparison of DNA polymerase/mismatch structures from diverse families shows the differences in the conformation of a particular mismatch depending on its location at the polymerase active site, the absence versus presence of a catalytic metal ion, and electrostatic environment of the active site.

**RMSD (Å)**	LIG1 EE/AA G:T PDB: 7SXE (present study)	LIG1 EE/AA A:C PDB: 7SX5 (present study)	LIG1 WT C:G PDB: 6P0C (ref: 30)	LIG1 EE/AA C:G PDB: 6P0D (ref: 30)	LIG1 EE/AA 8-oxoG:A PDB:6P0E (ref: 30)
LIG1 EE/AA A:T, PDB: 7SUM (present study)	0.598	1.046	0.801	0.744	0.812
LIG1 EE/AA G:T, PDB: 7SXE (present study)		0.736	0.766	0.688	0.740
LIG1 EE/AA A:C, PDB: 7SX5 (present study)			1.052	1.005	1.004

Supplementary Table 2: Comparison of RMSD values among LIG1 structures. Table represents the comparison of LIG1 structures (wild-type and EE/AA) in complex with the nick DNA containing cognate C:G or damaged 8-oxoG:A and our LIG1 EE/AA structures in complex with the nick DNA containing cognate A:T, mismatches G:T and A:C.

DNA Substrates	Sequence
Nick DNA with preinserted 3'-A:T	5’-CATGGGCGGCATGAACC**A**GAGGCCCATCCTCACC-3’-FAM<br>3’-GTACCCGCCGTACTTGG**T**CTCCGGGTAGGAGTGG-5’
Nick DNA with preinserted 3'-G:T	5’-CATGGGCGGCATGAACC**G**GAGGCCCATCCTCACC-3’-FAM<br>3’-GTACCCGCCGTACTTGG**T**CTCCGGGTAGGAGTGG-5’
Nick DNA with preinserted 3'-A:C	5’-CATGGGCGGCATGAACC**A**GAGGCCCATCCTCACC-3’-FAM<br>3’-GTACCCGCCGTACTTGG**C**CTCCGGGTAGGAGTGG-5’

Supplementary Table 3: Nick DNA substrates with 3'-FAM label used in the study. Nick DNA substrates were used in the ligation assays to test nick sealing efficiency of wild-type or EE/AA mutant of LIG1. FAM denotes a 6-carboxyfluorescein label and is located at 3'-end of the nick DNA substrates. The base at 3'-position is bold and the base at the template base position is underlined.

DNA Substrates	Sequence
One nucleotide gap DNA with template base C	FAM-5’-CATGGGCGGCATGAACC GAGGCCCATCCTCACC-3’-FAM<br>3’-GTACCCGCCGTACTTGGCCTCCGGGTAGGAGTGG-5’
One nucleotide gap DNA with template base T	FAM-5’-CATGGGCGGCATGAACC GAGGCCCATCCTCACC-3’-FAM<br>3’-GTACCCGCCGTACTTGGTCTCCGGGTAGGAGTGG-5’

Supplementary Table 4: One nucleotide gap DNA substrates used in the study. Gap DNA substrates were used in the coupled assays to test ligation of polβ nucleotide insertion products by LIG1. FAM denotes a 6-carboxyfluorescein label and is located at 5'- and 3'-ends of gap DNA substrates. The base at template base position is underlined.

DNA Substrates	Sequence
Nick DNA with preinserted 3'-G:T	FAM-5'-CATGGGCGGCATGAACC**G**GAGGCCCATCCTCACC-3'<br>3'-GTACCCGCCGTACTTGG<u>**T**</u>CTCCGGGTAGGAGTGG-5'
Nick DNA with preinserted 3'-A:C	FAM-5'-CATGGGCGGCATGAACC**A**GAGGCCCATCCTCACC-3'<br>3'-GTACCCGCCGTACTTGG<u>**C**</u>CTCCGGGTAGGAGTGG-5'

Supplementary Table 5: Nick DNA substrates with 5'-FAM label used in the study. Nick DNA substrates were used in the APE1 exonuclease assays. FAM denotes a 6-carboxyfluorescein label and is located at 5'-end of nick DNA substrates. The base at 3'-position is bold and the base at the template base position is underlined.

DNA Substrates	Sequence
Nick DNA with preinserted 3'-G:T	FAM-5'-CATGGGCGGCATGAACC**G**GAGGCCCATCCTCACC-3'-FAM<br>3'-GTACCCGCCGTACTTGG<u>**T**</u>CTCCGGGTAGGAGTGG-5'
Nick DNA with preinserted 3'-A:C	FAM-5'-CATGGGCGGCATGAACC**A**GAGGCCCATCCTCACC-3'-FAM<br>3'-GTACCCGCCGTACTTGG<u>**C**</u>CTCCGGGTAGGAGTGG-5'

Supplementary Table 6: Nick DNA substrates with 3'- and 5'-FAM labels used in the study. Gap DNA substrates were used in the coupled repair assays for APE1 exonuclease mismatch removal and ligation by LIG1. FAM denotes a 6-carboxyfluorescein label and is located at 3'- and 5'-end of nick DNA substrates. The base at 3'-position is bold and the base at the template base position is underlined.

Oligonucleotide	Primer	Sequence (5'-3')
1	Template T	GTCCGACTACGCATCAGC
2	Template C	GTCCGACCACGCATCAGC
3	Upstream A	GCTGATGCGTA
4	Upstream G	GCTGATGCGTG
5	Downstream	P-GTCGGAC

Supplementary Table 7: Oligonucleotide primers used for LIG1 crystallization. Oligonucleotides 1, 3, and 5 were used to prepare the nick DNA substrate with 3'-A:T. Oligonucleotides 1, 4, and 5 were used to prepare the nick DNA substrate with 3'-G:T. Oligonucleotides 2, 3, and 5 were used to prepare the nick DNA substrate with 3'-A:C. P denotes a phosphate group at 5'-end.
